# Socio-cultural predictors of health-seeking behaviour for febrile under-five children in Mwanza-Neno district, Malawi

**DOI:** 10.1186/1475-2875-8-219

**Published:** 2009-09-24

**Authors:** Alinafe I Chibwana, Don P Mathanga, Jobiba Chinkhumba, Carl H Campbell

**Affiliations:** 1 Malaria Alert Centre, College of Medicine, University of Malawi, P/Bag 360, Blantyre 3, Malawi; 2 Department of Community Health, College of Medicine, University of Malawi, Blantyre, Malawi; 3 CDC Malaria Malawi Programme, Centers for Disease Control and Prevention, Blantyre, Malawi

## Abstract

**Background:**

Prompt access to effective treatment for malaria is unacceptably low in Malawi. Less than 20% of children under the age of five with fever receive appropriate anti-malarial treatment within 24 hours of fever onset. This study assessed socio-cultural factors associated with delayed treatment of children with fever in Mwanza district, Malawi.

**Methodology:**

It was a qualitative study using focus group discussions and key informant interviews.

**Results:**

A total of 151 caregivers and 46 health workers participated in the focus group discussions. The majority of caregivers were able to recognize fever and link it to malaria. Despite high knowledge of malaria, prompt treatment and health-seeking behaviour were poor, with the majority of children first being managed at home with treatment regimens other than effective anti-malarials. Traditional beliefs about causes of fever, unavailability of anti-malarial drugs within the community, barriers to accessing the formal health care system, and trust in traditional medicine were all associated with delays in seeking appropriate treatment for fever.

**Conclusion:**

The study has demonstrated important social cultural factors that negatively influence for caregivers of children under five. To facilitate prompt and appropriate health-seeking behaviour, behavioral change messages must address the prevailing local beliefs about causes of fever and the socio-economic barriers to accessing health care.

## Background

Malaria remains the number one cause of fever and the leading cause of child mortality in sub-Saharan Africa [1] and presumptive treatment of fever in children with anti-malarial treatment is one of the key strategies for malaria control in this population [2]. Although the validity of providing presumptive treatment is now being called into question [3], there is no one who would argue against the principle of providing malaria treatment promptly. This is so because untreated falciparum malaria causes death within hours of the onset of symptoms [4], particularly among vulnerable groups, such as children under five years of age and pregnant women. Prompt access to treatment will become an even more important life-saving malaria control strategy if the trend in malaria transmission intensity continues to decline as has been documented in some parts of sub- Saharan Africa [5] and more people lose immunity for the disease. Roll Back Malaria has set a target of achieving at least 80% access to prompt and effective malaria treatment for children under five years of age by 2010 [6].

Currently, in many malaria endemic countries, coverage with prompt access to treatment remains low [7]. Factors that have been cited as responsible for the low coverage include the inability by caregivers to know the true cause of malaria [8,9], failure to link danger symptoms, such as convulsions and anaemia to malaria illness [10,5], and inappropriate diagnosis and prescriptions [11,12]. Even within a single country the factors can be complex [13] necessitating the need to consider local context in framing solutions. In Malawi, only 17% of the children receive anti-malarial treatment within 24 hours of onset of fever [14]. This is consistent with the World Malaria Report (2008), which showed that only 20% of the children in sub-Saharan Africa receive anti-malarial treatment within 24 hours of the onset of fever [2]. Few qualitative studies in the country have been done and these have largely focused on health-seeking behaviour and malaria treatment patterns [15,16]. There is paucity of country specific information on qualitative factors that influence prompt treatment. Therefore, the objective of this study was to explore community factors associated with delayed access to effective malaria treatment for children under the age of five years. Findings from this study will help in the development of behaviour change communication interventions to promote prompt access to effective anti-malarial treatment and will support the scale-up of such interventions in Malawi.

## Methods

This qualitative study explored factors affecting prompt access to malaria treatment in Mwanza-Neno, a rural district in southern part of Malawi. The district is served by 14 public health facilities and has a population of 203,373, 19.7% (40,064) of whom are children under the age of five years [10]. Malaria burden is high in the district with 50,000 clinical malaria episodes reported from health facilities annually. Usage of ITNs by children under-five is 20% and only 29.1% of children under-five had access to an effective anti-malarial drug within 24 hours in 2004 [17]. These indicators are low compared to national targets of 80%. Under five mortality rate (UFMR) is estimated to be 137/1,000 live births, which is higher than the national UFMR of 122/1,000 live births [18].

Data was collected through focus group discussions (FGDs) with both caregivers and health care workers and through in-depth interviews with key informants. For community discussions, FGDs composing 6-12 participants were conducted with women and men aged 15-49 years old who at the time of the study had under their care at least one child aged less than five years of age. To ensure free discussions, FGDs for men and women were done separately. Selection of study participants was done from eight randomly selected enumeration areas (census tracts with 150 - 300 households). Eight enumeration areas (EA) were deemed appropriate for the study to reach a point of saturation - i.e. when no more new information emerges from the data, and no new insights are generated. The study team and the district malaria coordinator visited the EA a day before the interviews to brief the village headman, village health committees and the community health workers about the study. On the day of interviews, eligible participants from the entire EA were invited to a meeting and briefed about the study and those willing to participate were recruited into the study. Efforts were made to include participants from across the entire EA. The FGDs were held in the local language by the Social scientist (Alinafe Chibwana), while two research assistants with expertise in public health, sociology, and medicine assisted by tape recording the discussions and taking down notes.

Health workers involved in the FGDs were recruited from six randomly selected health facilities. This included clinical officers, nurses, community health workers, and hospital attendants. Since there is no inferential statistics in qualitative research, the number 6 constituted the experiences, knowledge and attitudes sufficient enough to tackle the issues in question. These health workers were selected purposefully based on their involvement in malaria treatment and prevention. On the day of the FGD, the health facility was visited by the study team and all eligible health workers were encouraged to participate. In-depth interviews were also conducted with the District Malaria Coordinator (DMC) and the District Health Officer (DHO) as key informants (KIs), selected purposively considering their role as members in the District Health Management Team (DHMT).

Ethical approval for the study was obtained from the Institutional Review Boards at the University of Malawi and the U.S. Centers for Disease Control and Prevention.

### Data analysis

Thematic analysis was the major framework for analyzing the data. Data recorded on the tapes during the focus group discussion and in-depth interviews were listened to repeatedly in order to get familiar with the data. There was a comparison of words, emphasis of the respondents' comments, consistency of comments, and the specificity of responses in follow-up probes. Similar thoughts expressed across the participating focus group discussions were identified, coded and grouped together. Out of each group of similar thoughts, a unifying concept or underlying theme was derived. Key points, catch phrases and illustrations were also identified to back up the findings. Finally, emerging themes that were similar were grouped together to come up with major themes through a consultative process among authors.

## Results

### Social demographic characteristics of participants

Twenty-one focus group discussions (eight FGDs with women, seven FGDs with men and six FGDs with health workers) were done. Two in-depth interviews with the district malaria coordinator and District Health Officer as key informants were also held. A total of 197 respondents participated in this study (94 women, 57 men, 44 health workers and two KII).

Out of 94 women who participated, 6% were below 20 years of age, 92% were aged between 20 and 40 years and only two women were more than 40 years old. Most women (81%) had at least some education at primary or secondary school level and majority (88%) were involved either subsistence farming or small-scale businesses. The male participants were older with 80% aged between 20 and 40 years and 18% aged more than 40 years old. Just like the women, most men were involved either in subsistence farming or small scale business. Out of 44 health workers involved in FGDs, 13 were nurses, six were clinicians, five were medical assistants, 13 were community health workers and seven were hospital servants.

The possible sources of malaria treatment available to these communities included traditional healers, small groceries, the rural health centre, few private clinics and fellow friends with left over drugs from a previous illness.

Results are presented thematically below while Additional File 1 summarizes these findings.

### Knowledge of the causes of fever and danger signs of malaria

Ability to recognize fever and associate it with malaria was very high amongst the caregivers. Fever or hot body meant that a child has malaria. However, when participants were directly asked what caused the fever in under five children, both male and female caregivers listed non-fever causes. This included poor sanitation in the home, *cham'mimba *which refers to uterine contraction during delivery, coldness, sleeping without a blanket, soaking in the rains, malnutrition, playing in the dust, not bathing the child, eating un-boiled food, playing in water and a result of falling down during play. Malaria, pneumonia, immunization, headache, cough, sneezing, teething, persistent crying, diarrhoea, anaemia, blisters in legs or arms, known as *mwana mphepo *in local language, and acute respiratory infections were also mentioned as causes of fever in children. There were strong beliefs also that mother's reproductive tract illnesses locally called mauka were responsible for fever in under-five children. Out of 15 FGDs with caregivers, 10 FGDs mentioned pathological vaginal growths such as warts and six FGDs mentioned vaginal itching, (*mauka*), in the mothers as being the cause of fever in under-five children.

One woman said:

"*According to our traditional beliefs, fever in children under-five years old is due to various types of mother's reproductive system disorders such as vaginal itching, vaginal discharge and genital warts*."

One man said:

"*If a child has fever on and off, we call it 'Mauka'. The fever may disappear in the morning, then resurface in the afternoon and fever cools down at dawn."*

Health care providers similarly mentioned that majority of caregivers recognize fever. It was mentioned that most women in rural areas associate fever with malaria and also link fever to mother's reproductive tract illnesses such as vaginal growths and discharge. As a result, most caregivers were of the opinion that childhood fever could only be treated if the mother's illness was appropriately treated first either by traditional healers or at the local health facility (See Table 1: Causes of fever mentioned by caregivers).

**Table 1:** Causes of fever mentioned by caregivers

**Cause**	**Frequency by women (No of FGDs N = 8)**	**Frequency by men (No of FGDs N = 7)**
Malaria	8	7

Cough	6	1

Teething	5	1

Sneezing/fluenza	3	1

Pneumonia	2	2

Anemia	2	0

Immunization	1	0

Diarrhoea	1	0

Other diseases	0	1

Vaginal growth (warts)	6	4

Vaginal itching	4	2

Coldness	4	5

Vomiting	4	0

Persistent crying	3	0

Malnutrition	2	2

Not bathing the child	1	2

Headache	1	2

Falling down during play	1	1

Cloudy atmosphere	1	1

Poor sanitation in the home	0	1

Improper flow of blood	1	0

Playing in the dust	1	0

Playing in water	1	1

Eating unboiled food	0	1

Soaked in rains	0	1

Mwana-mphepo	2	1

Cham'mimba	2	0

Majority of caregivers mentioned that apart from fever, vomiting, diarrhoea, lethargy, refusing to suck, refusing to eat, irritability, bulging fontanelle, rash, rigors, coughing, groaning and sunken eyes, were signs and symptoms they consider to diagnose that a child has malaria. Most of caregivers mentioned high fever, lethargy, refusing to eat/suck, frequent vomiting and diarrhoea as major signs of malaria. Other danger signs reported in few FGDs include bulging fontanelle, weak eyes, rigors, groaning, irritable, rash, pneumonia and mother's reproductive tract illnesses. Ignorance of danger signs of malaria was high with very few participants mentioning convulsions and anaemia as danger signs. Of all 15 FGDs with caregivers, only two had members who were able to link anaemia with malaria.

One woman said, *"I go to the hospital same day of onset of fever because we fear that the child would become anemic if we don't treat him faster"*

None of the FGDs, except one, linked convulsion to malaria. Convulsions and in some FGDs, anaemia, was attributed to other causes, such as epilepsy and witchcraft respectively.

One woman said, *"I was just giving my child panadol because they didn't find any problem with the child at the hospital. However, things worsened when I went home as the child started convulsing and I thought it was epilepsy. We gave him herbs but did not respond favorably. Then we decided to go to the hospital, the doctor said, the child had been ill for quite some time and this was not good to bring a child for medical attention too late."*

One medical assistant attested, "*I have referred a child today who has been convulsing and the mother thought that it was epilepsy or witchcraft yet it was malaria"*.

### Treatment patterns for fever

Most caregivers reported that they manage fever at home by tepid sponging; bathing the child with cold water, give water frequently, visit the herbalist to cure '*mauka*', covering the child with warm clothes and giving antipyretics, such as paracetamol. The coverage with effective malaria treatment is much lower, only one FGD with the women mentioned that they give Sulphadoxine-pyrimethamine before they go to the hospital. They acknowledged that treatment for malaria with any anti-malarial drug was given after more than 48 hours, because in the first 24 hours antipyretics were given as first aid for fever and were viewed as effective treatment for fever.

One woman said, *"We give our children panadol because it is what we are given when we go to the hospital with a child suffering from malaria."*

According to health workers, when mothers were asked for drugs they administered to children before seeking treatment at a health facility, majority of the mothers mentioned antipyretics and antibacterial treatment. Anti-malarial treatment, such as Sulphadoxine-pyrimethamine was only given when the child's symptoms did not improve following the use of antipyretics and anti-bacterials.

A clinician at Neno rural hospital said, *"Women say, we gave him/her panadol thinking that it cures malaria."*

For those who believed that fever was caused by the mothers' illness, traditional healers were their first source of treatment for fever:

One man said: *"If a child has fever on and off we call it mauka (vaginal growths) and the best remedy is having those warts removed by the traditional healer and the fever in the child will disappear 3 to 5 days after removing the mauka"*.

Health care providers confirmed that caregivers' belief in traditional healers was one of the reasons distracting attention from prompt effective treatment of malaria as reported by one clinician:

"*So many times, I see fresh tattoos on children's bodies as I examine them which confirm a visit to a traditional healer."*

### Poor access to anti malarial drugs at the community level

In all the FGDs, lack of access to anti-malarial drugs was mentioned as one of the problems that prevented caregivers from giving the needed drugs promptly. Financial constraints to buy anti-malarial drugs, inadequate knowledge on the correct dosage, and fear of giving expired drugs from shops and distance to health facilities were some of the main reasons given for failing to provide an effective anti-malarial treatment within the home.

One woman said, *"It is a long distance to the health facility, so we give panadol purchased from the shops first and wait for the child to improve. We go to the hospital when there is no improvement. There are no anti-malarial drugs in the community except for left over drugs given from the hospital sometimes from a previous episode"*.

### Perceived severity of fever

Caregivers had their own way of categorizing fever into mild and severe. In general, children with fever who are able to play were classified as having mild fever whereas febrile children who could not play were considered as having severe fever. Most caregivers did not appreciate the potential harm of mild fever, hence the delay in starting appropriate treatment. This was corroborated by health workers who believed that caregivers only sought effective treatment when life-threatening symptoms and signs were noticed. Health workers from all facilities said that 30% of under- five children only sought treatment from public health facilities when severe signs such anemia, convulsions, respiratory distress and cerebral malaria had developed.

One clinician said, *"Most women say that their child has been ill since dzana (three days ago) and they come to the hospital with bad cases like complications of anaemia and you know that it is not dzana but sometime back"*.

This is evidenced from FGDs with women when they were asked time they take to treat children from onset of fever. Majority of the women admitted that treatment was only sought from health facilities more than 48 hours after symptoms developed or only when symptoms were considered severe.

One caregiver said, *"It depends on severity of fever, if it is not severe, we stay. When we have given the child painkillers and the fever goes down within 2 to 3 days, we don't go to the hospital and we conclude that it was not malaria, but attribute this to something else like food poisoning"*.

### Health facility characteristics as barriers to prompt treatment of fever

Health facilities promote long queues due to the design of the system which mixes adults, youths with under five children and due to the fact that there is no specific clinician for under five children. The facilities close at 12 o'clock, for a lunch break, leaving behind a number of children unattended. Further, the facilities are closed during weekends and sometimes the medical assistant is not around.

"I visited the hospital very early and when I saw the clinician coming, I thought he wanted to open the hospital, only to be told that go and buy drugs in the shop, am going to Mwanza boma."

Health workers and caregivers also mentioned other facility-based factors such as shortage of drugs and lack of diagnostic capacity as reasons for not seeking care promptly.

One woman said, *"We get demotivated after walking to the health center, waiting for treatment and only to be told that drugs are out of stock"*.

Some caregivers complained of mistreatment by health workers as reasons for not seeking care at health facilities. They reported that there is no triage at the hospital a result some children die while on the queue and due to long queues clinicians make hasty examinations, missing out some relevant information for appropriate treatment.

Another woman said, "*They question, 'where were you in the morning?' Yet we don't plan that a child falls sick in the afternoon, and are shouted at badly"*

During health worker FGDs, health workers acknowledged that there is usually a shortage of staff, which negatively affects their attitude towards care seekers.

One clinician said, "*Just imagine, we sometimes see over 120 patients a day and we get tired and we may speak badly in the process."*

### Lack of women empowerment in decision making

The majority of women caregivers felt that they were not empowered to make decisions affecting positive health-seeking behaviour independent of their husbands or other family members. Health workers and caregivers highlighted that the absence of family heads or other key decision makers from households often contributed to delays in seeking care.

Most women reported that their health-seeking behaviour is very poor because men usually don't respond when a child has fever; they respond only when a child's condition worsens.

One woman said, *"Men are not moved with fever but when they see the child is very sick, breathless and your facial expressions have changed, that's when they panic and take a step to seek medical attention."*

Men in FGDs reported that they are always busy and have no time to attend to a sick child and women fail to decide to seek medical attention.

One man said, *"When the women say the child has fever, we tell them to buy panadol for the child. Thereafter, we go on to attend to other activities; we get to see the child later*".

A medical assistant said, "One *woman came with a very sick child and when asked about the delay she said that she was waiting for approval from her uncle who was in Mozambique by then."*

## Discussion

Despite increasing investment in malaria control, prompt access to effective anti-malarial treatment remains unacceptably low in many malaria endemic countries, including Malawi [2,9,19]. A variety of interconnected factors at the household and health system level influence access to prompt and effective treatment of fever. Furthermore, access to prompt treatment may be influenced by global and national health policies [2]. Some of these factors vary from setting to setting and there is need to consider local context in framing intervention strategies. This study describes socio-cultural factors such as local beliefs about causes of fever in children, perceived severity of childhood fever, strong belief in traditional healers, limited availability of anti-malarial drugs at the community level, and health facility-based characteristics, such as distance and mistreatment by health workers, are important deterrent factors preventing many caregivers from seeking prompt malaria treatment for children under five in the country.

The clinical presentation of malaria is highly variable and in addition to fever, may include chills, sweats, vomiting, nausea and backache to meet the biomedical definition of malaria though the term is not compatible with biomedical definition of malaria[13]. In Malawi, the local name for malaria is "*malungo*". However, for lack of better local word to describe fever, a person with fever is often said to have "malungo" [20]. It is not surprising, therefore, that even though fever was largely linked to malaria the responses were varied and not always associated with specific treatment for malaria. It was also noted that in those caregivers that clearly linked fever to malaria, the treatment they provided was not always appropriate. Majority of them either administered drugs such as antipyretics (panadol) other than anti-malarials or consulted traditional healers for treatment of childhood fevers.

Fever that was categorized as "mild" was more likely to be self treated in the homes while treatment for "severe" fevers was often sought from outside the homes, commonly from the formal health care system. Treatment-seeking behaviour based on perceived severity of fever is in line with other findings from the region [9,21,22]. The notion that some fevers are "mild" and do not merit early treatment compared to "severe" fever has important ramifications for malaria control in children, as malaria can kill swiftly, often within hours of the onset of fever symptoms [1]. This finding is cause for concern in Malawi considering that some caregivers did not know the dangerous symptoms and signs of malaria. Future malaria campaigns should, therefore, emphasize the clinical importance of all kinds of childhood fevers irrespective of local grading of fever severity.

Local beliefs regarding aetiology of fever strongly influenced health-seeking patterns. For instance, caregivers are likely to consult traditional healers when they believe that childhood fever is due to witchcraft or are linked to maternal ailments, such as *mauka*. In almost all FGDs, traditional healers were mentioned as the first resort for 'mauka' related childhood fever since it was believed that the mother's illness had to be treated first. This interest in traditional healers in managing fever at community level is in sharp contrast with local community surveys, which have shown that less than 1% of childhood fever sought help from the traditional healers [9]. The possible explanation for this disparity is that respondents in surveys may primarily refer to non-mauka related fever for which care is sought at the health care facilities where as respondents to qualitative studies refer mauka related fevers. Nonetheless, this interest in traditional healers provides an opportunity for collaboration between the traditional healers and the formal health system in increasing prompt access to effective treatment for malaria. Historically, traditional healers have often been thought of as being harmful in malaria treatment and prevention since they appear to delay patients from accessing appropriate treatment. However, in Tanzania, the involvement of traditional healers led to an improved early referral for children with severe malaria and prompt treatment [6]. Deliberate efforts should be made to include and train traditional healers in malaria control either as community dispensers of anti-malarial drugs or to counsel caregivers regarding the need for appropriate and early referral to a health facility.

The study also shows that most childhood fevers are first treated outside the formal health care system, where some health facility-based attributes, such as distance, quality of care and availability of drugs, are some of the reasons for seeking care outside the formal health care system. This treatment-seeking behaviour is supported by previous surveys in Malawi, which have shown that about 60% of children with fever are first treated within the community [9] and is not uncommon in sub-Saharan Africa [22,23]. These findings highlight the importance and role of the home-based management of fever (HBMF) strategy in promoting access to prompt treatment of fever in children and malaria control. Whilst HBMF is being promoted by the World Health Organization [24], most endemic countries do not have the systems in place to support a successful HBMF strategy. In Malawi, artemisinin-based combination treatment (ACT) for malaria treatment is only available at health facility level. This limited availability of ACT is in marked contrast to the previous first line treatment with SP, which until recently was readily available at community level for HBMF. This unavailability is compounded by health facility access problems, such as distance and the various barriers highlighted by caregivers in accessing treatment for malaria fever. Therefore, it is crucial that national malaria programmes roll out free or highly subsidized ACT at community level to close the existing gap in the availability of effective drugs and to increase prompt access to effective anti-malarial treatment.

## Conclusion

This study has shown that future successful community mobilization for prompt and effective treatment for childhood fevers are more likely to be achieved if IEC campaigns address local beliefs about fever and malaria and efforts are made to work with health care providers outside the formal health system. It is also important that anti-malarial drugs be made widely available within the community to enable HBMF to become a successful intervention. This is especially important in countries, like Malawi, where the majority of fevers are first treated within the community. Finally, it is also important that malaria control interventions to promote prompt access to appropriate and effective treatment start recognizing and addressing other perceived and real barriers to health-seeking behaviour, such as the cost of anti-malarial drugs in the private sector and community, distance and transportation costs to health facilities, health provider staff shortages, and empowerment of women as decision-makers at the household level.

## Conflict of interest statement

The authors declare that they have no competing interests.

## Authors' contributions

AC contributed to the study design, fieldwork, data analysis, interpretation, drafting and revision of manuscript. DM was responsible for study conception, data interpretation, revision and final approval of the manuscript. JC took part in interpretation of results, revision of draft and final manuscript. CC obtained funding, contributed to the data interpretation and drafting, revision and final approval of the manuscript.

## Supplementary Material

Additional file 1 **Findings and Illustrations from all FGDs and KIs**. This file highlights findings from all Focus Group Discussions and in-depth interviews with caregivers and health care providers. Findings, themes and important quotes or illustrations are provided. Click here for file
